# Ventricular tachycardia in ischemic cardiomyopathy; a combined endo-epicardial ablation as the first procedure versus a stepwise approach (EPILOGUE) – study protocol for a randomized controlled trial

**DOI:** 10.1186/s13063-015-1005-6

**Published:** 2015-10-29

**Authors:** Astrid A. Hendriks, Muchtiar Khan, Laszlo Geller, Attila Kardos, Lennart J. de Vries, Sing-Chien Yap, Sip A. Wijchers, Dominic AMJ Theuns, Tamas Szili-Torok

**Affiliations:** Department of Clinical Electrophysiology, Erasmus Medical Center, Postbus 2040, 3015 CE Rotterdam, The Netherlands; Department of Clinical Electrophysiology, Onze Lieve Vrouwe Gasthuis, Amsterdam, The Netherlands; Department of Clinical Electrophysiology, Cardiovascular Center Semmelweis University, Budapest, Hungary; Department of Clinical Electrophysiology, Hungarian National Institute of Cardiology, Budapest, Hungary

**Keywords:** Epicardial ablation, Ischemic cardiomyopathy, Ventricular tachycardia

## Abstract

**Background:**

The role of epicardial substrate ablation of ventricular tachycardia (VT) as a first-line approach in patients with ischemic heart disease is not clearly defined. Epicardial ablation as a first-line option is standard for patients with nonischemic dilated cardiomyopathy and arrhythmogenic right ventricular cardiomyopathy. Several nonrandomized studies, including studies on patients with ischemic heart disease, have shown that epicardial VT ablation improves outcome but this approach was often used after a failed endocardial approach. The aim of this study is to determine whether a combined endo-epicardial scar homogenization as a first-line approach will improve the outcome of VT ablation.

**Methods/Design:**

The EPILOGUE study is a multicenter, two-armed, nonblinded, randomized controlled trial. Patients with ischemic heart disease who are referred for VT ablation will be randomly assigned to combined endo-epicardial scar homogenization or endocardial scar homogenization only (control group). The primary outcome is recurrence of sustained VT during a 2-year follow-up. Secondary outcomes include procedural success and safety.

**Discussion:**

This study is the first randomized trial that evaluates the role of a combined endo-epicardial scar homogenization versus endocardial scar homogenization for the treatment of ischemic scar-related VT.

**Trial registration:**

NL4816807814v02

## Background

Ventricular tachycardia (VT) is an important clinical sequela after a myocardial infarction and may be associated with sudden cardiac death [1]. The introduction of the implantable cardioverter defibrillator has provided an important tool in the prevention of sudden cardiac death. However, a significant proportion of patients experience multiple appropriate implantable cardioverter defibrillator shocks due to sustained VT. Catheter ablation of VT is useful to reduce the burden of VT. The threshold for VT ablation has lowered in recent years, mainly as a result of technological advancements and increased experience [2–4]. The endocardial approach has been the preferred first-line approach for VT ablation in patients with ischemic heart disease.

Recent studies suggest that 12–17 % of all VTs in a mixed population of ischemic and nonischemic cardiomyopathies have an epicardial origin [5, 6]. Epicardial VTs have been observed in 10 % of post-infarction VTs [7]. These numbers may be underestimated, considering the moderate outcomes of endocardial VT ablations [8, 9]. In experienced centers, epicardial VT ablation has an acceptable risk-to-benefit ratio [6, 10]. Current recommendations for scar-related VT suggest that patients with ischemic heart disease can benefit from an epicardial approach if an endocardial ablation fails [11].

The aim of this randomized trial is to determine whether combined endo-epicardial scar homogenization as a first-line approach will improve the outcome of VT ablation in comparison with endocardial scar homogenization only.

## Methods/design

### Study design

**Fig. 1:**
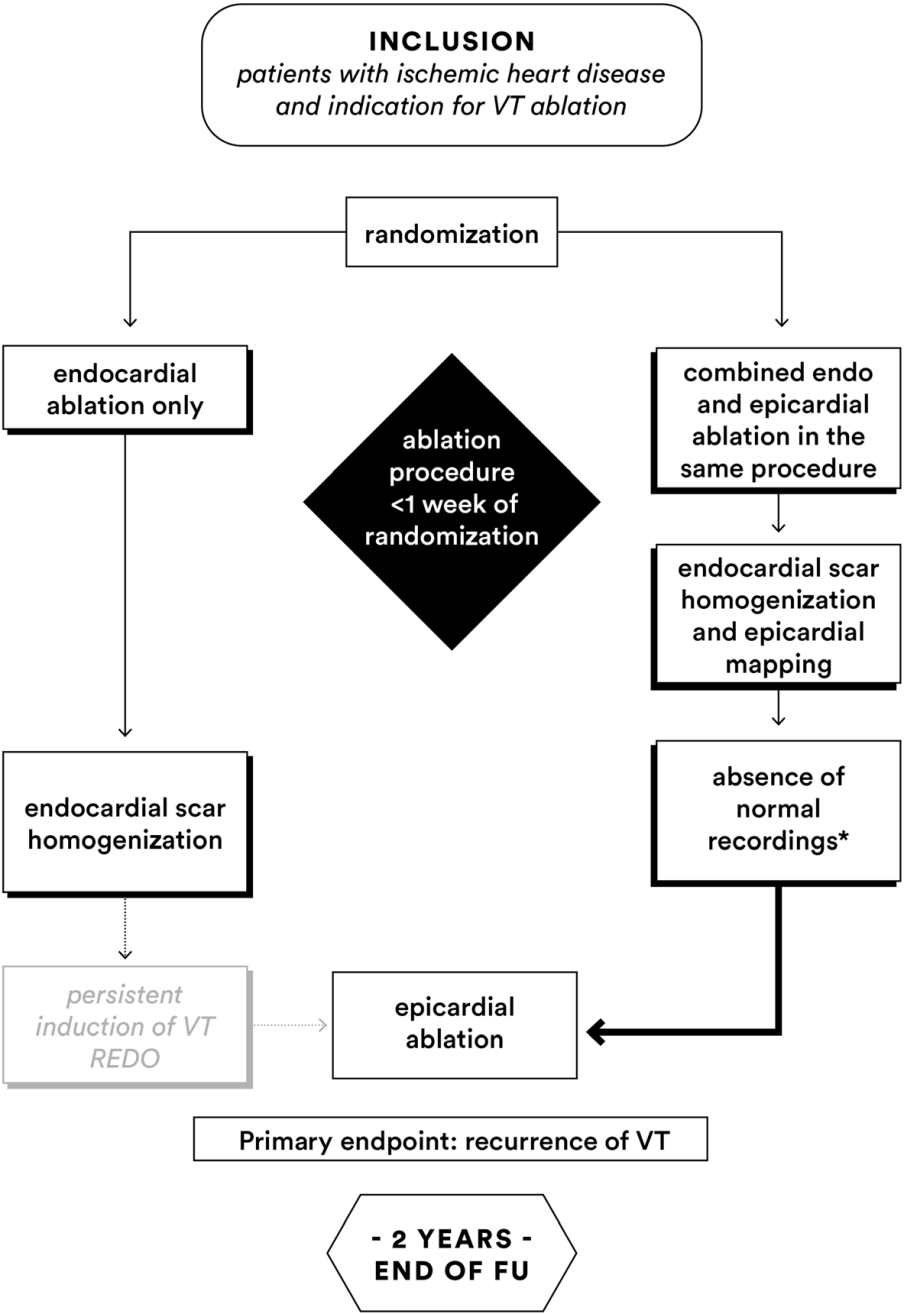
EPILOGUE study flow chart. *Normal recordings are defined as not more than three sharp and discrete deflections from baseline, amplitude >1.5 mV, duration >70 ms or amplitude-to-duration ratio >0.046. FU, follow-up; VT, ventricular tachycardia

The EPILOGUE study is a multicenter, two-armed, nonblinded, randomized controlled trial. Patients will be randomized to endocardial scar homogenization or endo-epicardial scar homogenization (Fig. 1). The study period is 2 years after the index procedure. All participating centers are tertiary referral centers for VT ablation with experience in epicardial ablation.

### Study population

All patients with a history of ischemic heart disease who are referred for VT ablation will be considered for inclusion. Patients can be included if they meet all of the following inclusion criteria:

- Age ≥18 years;
- History of ischemic heart disease;
- Presence of or planned-for implantable cardioverter defibrillator soon after VT ablation.

Patients will be excluded if they meet any of the following criteria:

- Presence of unstable angina;
- Acute myocardial infarction <30 days (or in case of incessant VT, <14 days);
- Absence of visualization of coronary anatomy (coronary angiogram or computed tomography);
- Presence of significant coronary stenosis clinically relevant for intervention;
- Presence of mobile left ventricular thrombus;
- Presence of aortic or mitral mechanical valve prosthesis;
- Previous pericarditis;
- Previous coronary artery bypass graft;
- Other factors that could potentially lead to pericardial adhesion (e.g., thoracic radiation therapy);
- Contra-indication for general anesthesia.

### Ethics

The study protocol was approved in February 2015 by the Medical Ethical Committee (2014–248) Erasmus Medical Center, Rotterdam, The Netherlands. Informed consent will be obtained from each participant.

### Primary and secondary outcomes

The primary outcome of this study is recurrence of sustained VT (defined as appropriate implantable cardioverter defibrillator therapy or VT >30 s duration) within 2 years. We will use a blanking period of 1 week after the ablation procedure. The secondary outcome parameters will be acute procedure success and procedure-related major complications. Procedural success will be defined as complete success if there is noninducibility of any sustained monomorphic VT. Partial success will be defined as noninducible clinical VT but inducible polymorphic VT, or VT with a cycle length <200 ms. Procedural failure will be defined as a spontaneous occurrence of VT or inducible clinical monomorphic VT.

Furthermore, the following data will also be determined: procedure time, fluoroscopy time, radiofrequency ablation time, time to recurrence of the ventricular arrhythmia, number of appropriate implantable cardioverter defibrillator therapies on follow-up, number of VT-related hospitalizations, freedom of antiarrhythmic drugs on follow-up, repeated (epicardial) ablation procedures, evidence of an incessant VT or VT storm during follow-up, and mortality.

### Study groups

This study has two study arms, the endocardial approach only and the combined endocardial and epicardial approach (combined approach). Crossover is not allowed during the first procedure. When a patient is randomized to the combined approach and the epicardial access is unsuccessful, this is considered a screening failure.

### Randomization

Patients are randomized to either the endocardial approach or the combined endocardial and epicardial approach. Randomization is performed using a computer-generated program (ALEA). The person carrying out randomization will not be the treating physician or involved in the selection procedure. The patient and the electrophysiologist who will be performing the procedure will not be blinded for the method of approach.

### Electrophysiology procedure and catheter ablation

#### Pre-procedural preparation

In general, anticoagulation therapy will be discontinued before the procedure (unless the operator decides otherwise). When performing the pericardial puncture, the international normalized ratio should be <2.0. Transthoracic echocardiography will be used to exclude a thrombus in the left ventricle. If the transthoracic echocardiography is inconclusive, contrast transthoracic or transesophageal echocardiography will be used. Antiarrhythmic medications will be stopped 5 days before the procedure. In case of an emergency VT ablation, the operator can choose to do the ablation while continuing antiarrhythmic drugs. Patients who undergo an epicardial ablation will receive general anesthesia; otherwise conscious sedation and local anesthesia will be used. The epicardial ablation is performed with a surgical team as backup.

#### Pericardial access

In patients undergoing epicardial ablation, before ablation and systemic heparinization, subxiphoid pericardial access will be obtained by fluoroscopic guidance (lateral plane). Using a Tuohy needle, small amounts of contrast are injected while carrying out the puncture. Guided by fluoroscopy, a guide wire is inserted into the pericardial space and an Arrow sheath is placed. An appropriate sized guiding catheter is inserted to keep the sheath open. During ablation, we will use an irrigated tip catheter. The baseline flow should be reduced to 1 ml/min. Fluid will be drawn from the pericardial space frequently during the ablation.

At the discretion of the operator, epicardial phrenic nerve capture will be performed if the origin of the VT is located in the area of the phrenic nerve. When capture is performed, a balloon is inserted and inflated to avoid phrenic nerve damage during ablation.

At the end of the procedure, a pericardial drain is inserted for 24–48 h. The operator will administer an intrapericardial steroid injection. Colchicine should be prescribed for up to 1 month, commencing on the first day after the procedure. The first day post procedure Colchicine is dosed 1 mg twice daily followed by a maintenance dose of Colchicine 0.5 mg twice daily.

#### Mapping

Endocardial mapping will be performed in all patients. A multipolar diagnostic catheter will be placed in the coronary sinus. A quadripolar diagnostic catheter will be introduced via the femoral vein to the right ventricular apex. Left ventricle mapping will be performed via the retrograde aortic or transseptal approach. The procedure will be performed under intravenous anticoagulation with heparin, with a target activated clotting time >250 s.

Electroanatomical maps will be obtained using CARTO 3 (Biosense Webster, Diamond Bar, CA, USA) or EnSite NavX (St. Jude Medical, St. Paul, MN, USA). This includes activation (if applicable) and voltage maps. The bipolar voltage thresholds used to consider dense scar and border zone will be 0.5 and 1.5 mV, respectively. We will aim to acquire a very dense map, especially around scar areas. Areas of fractionated or late potentials will be annotated. Programmed ventricular stimulation will be performed, and will involve three extra stimuli from two different right ventricular sites, high-rate pacing, and intravenous isoproterenol (at the discretion of the operator). In hemodynamically unstable VTs mechanical circulation support (Impella, intra-aortic balloon pump) can be considered.

#### Ablation protocol (both groups)

If a hemodynamically tolerated VT is induced during the electrophysiological study, a standard activation or entrainment mapping is carried out. The ablation will continue until the VT is no longer inducible. Additionally, endocardial scar homogenization will be performed. Based on the substrate map, ablation will be empirically extended throughout the entire scar endocardially. Also delayed and fractionated electrograms will be targeted for ablation. If clinical VT is still inducible in the endocardial ablation-only group, an epicardial VT ablation will be performed in a ‘redo’ procedure. Epicardial mapping and ablation will be performed as described in the combined approach.

#### Combined endocardial and epicardial group ablation

After obtaining pericardial access, a substrate map is constructed to identify abnormal electrograms. Activation mapping during VT – if possible – is done to prove epicardial involvement. Normal electrogram recordings show not more than three sharp and discrete deflections from baseline, amplitude >1.5 mV, duration <70 ms or amplitude-to-duration ratio >0.046 [9]. Before epicardial ablation, a coronary angiogram will be performed to avoid coronary artery damage. In addition to targeting abnormal potentials, epicardial homogenization will be performed. Ablation is continued until all abnormal potentials are gone.

### Clinical outcome and follow-up

Patients will be seen periodically up to 2 years (at 1, 3, 9, 12, and 24 months) after the index procedure. All implantable cardioverter defibrillator therapies and clinical events are reported.

The implantable cardioverter defibrillator will be programmed to detect VTs slower than the clinical VTs. Recommended implantable cardioverter defibrillator programming consists of a ventricular fibrillation zone with a cut-off rate of 220 beats per minute and a therapeutic VT zone with a cut-off cycle length of 60 ms below the slowest documented VT. To register VTs with rates below the detection limit, a monitor zone will be programmed with a cut-off rate of 120 beats per min. Detection duration will be 24/32 beats or 5 s in the ventricular fibrillation zone and 26 beats or 10 s in the therapeutic VT zone.

The analysis of recurrence of VT depends on the clear differentiation of VT from supraventricular tachycardia events. For reliable adjudication of arrhythmic events, implantable cardioverter defibrillator episodes with available electrograms are mandatory. In the case of single-chamber devices, the recommended setting is storage of both near-field and far-field (shock) electrogram sources. In addition, electrogram storage must be available before arrhythmia onset.

### Adverse events

In compliance with medical ethics committee regulations, it is compulsory to register all serious adverse events. Death, acute myocardial infarction, coronary artery damage, type III and V major bleeding, abdominal bleeding, tamponade of more than 80 cm^3^, late tamponade, and an ischemic cerebral event are considered major adverse events. Dry right ventricle puncture, drainable hemopericardium, postprocedural precordial pain, phrenic nerve injury and type II minor bleeding [12] are considered minor adverse events.

### Safety monitoring

Two committees and one monitor will be appointed to monitor patient safety. A data safety monitoring board will review study progress and has the authority to end the study if significant benefits or adverse effects in either study arm are suspected. The board members are three experienced electrophysiologists and a statistician. The clinical events committee will study all serious adverse events for a causal relationship between events and the EPILOGUE study. A monitor will evaluate protocol compliance by taking random samples in all participating centers.

### Statistical analysis

Continuous variables between groups will be compared using Student’s *t* test or the Mann–Whitney test for continuous variables, where appropriate. Categorical variables between groups will be compared with the chi-square test or Fisher’s exact test, where appropriate. The primary outcome parameter will be analyzed using Kaplan–Meier survival curves and tested with a log-rank test. A two-sided *P* value of 0.05 will be considered statistically significant for all analyses. Statistical analysis will be performed using SPSS version 20.

### Sample size calculation

Sample size was calculated based on the following assumptions: a 29 % difference in primary outcome event rate (recurrence of VT) between the endocardial and epicardial group (15 %) and the control group (44 %) after a follow-up time of 2 years [9]. We calculate that a sample size of 86 patients is required to achieve an 80 % power to detect a significant difference with a (two-tailed) alpha error of 5 %. Allowing for a dropout rate of 10 %, we calculated that we would require 100 subjects.

## Discussion

### Mechanism of scar-related ventricular tachycardia

Sustained VT in the presence of coronary artery disease is most often the result of prior myocardial infarction [13]. Scar areas form the substrate for macro-reentry which is the most frequent mechanism underlying VT. Targets for ablation are the area of low bipolar voltage that corresponds to the subendocardial projection of the scar, and potential targets within the scar that represent critical diastolic isthmuses during VT.

### Role of epicardial ablation

In 73 % of unsuccessful endocardial ablations, evidence of an epicardial substrate of VT was found [14]. To reach procedural success in VT ablation, Cesario et al. [15] found that 40 % of the patients were in need of an epicardial ablation. A recent retrospective observational study from Tung et al. [16] compared the outcomes of patients who underwent epicardial ablation with the outcomes of patients who did not. The pericardial space could not be accessed in 7 % by the percutaneous technique. The study demonstrated that at 12 months those with an ischemic substrate for VT who underwent a combined endocardial and epicardial ablation had improved freedom from VT compared with those who underwent endocardial-only ablation (85 % versus 56 %; *P* = 0.03). In a multicenter study by Della Bella et al. [5], 67 % of a subgroup of patients with ischemic VT etiology were in need of a stepwise approach. It could be speculated that an endocardial and epicardial approach as a first-choice ablation strategy in this subgroup might have avoided a substantial number of repeat procedures and improved outcome.

### Epicardial ablation in ischemic cardiomyopathy

Multiple studies on epicardial VT ablation in scar-related VT have been published [5, 9, 15, 17, 18]; however, few have compared endocardial ablation with a combined endocardial and epicardial ablation.

In 33 post-infarct patients [17], epicardial mapping was performed when endocardial ablation failed. These 33 patients were retrospectively compared with patients who had had an endocardial ablation only. There was found to be no difference in outcome. However, only 6 % of the patients had had an epicardial ablation.

A recent study from Di Biase et al. [9] compared limited endocardial substrate ablation with combined endocardial and epicardial scar homogenization in patients with an electrical storm and ischemic cardiomyopathy. There was a lower VT recurrence rate in those who underwent endocardial and epicardial scar homogenization during a mean follow-up of 25 months (19 % versus 47 %; *P* = 0.006). A limitation of this study is that because these researchers used a historical control group, the technique of ablation (limited substrate ablation versus scar homogenization) differed between groups.

### Safety of epicardial ablation

The reported complication rates associated with epicardial ablation range from 0.6 to 4.1 % [5, 6]. Procedure-related death in most studies was 0 % [5, 6, 16]. However, a study from Hamburg reported two procedure-related deaths in 59 patients undergoing epicardial VT ablations [14]. Thus, life-threatening complications might occur. Other reported major complications were [5, 6, 9, 16]:

- Hepatic bleeding;
- Pericardial bleeding requiring a drain;
- Phrenic nerve palsy;
- Pleuro-pericardial fistula;
- Coronary artery damage;
- Cardiac tamponade;
- Pericardial inflammatory reaction.

To prevent pericardial inflammation and subsequently adhesions, the use of colchicine after epicardial ablation is mandatory in our study [19].

### Study design

The study of Di Biase et al. [9] was a retrospective comparison of two techniques. Although there was a well-written prospective protocol, the two well-known techniques are difficult to compare. In the absence of a large scar or in the presence of dense scars, patients did not undergo epicardial ablation. Only 14 out of 43 patients (33 %) in the endocardial and epicardial scar homogenization group underwent epicardial VT ablation. In the current study both groups will undergo scar homogenization. Patients randomized to the combined approach will undergo epicardial ablation unless there is a complete absence of abnormal recordings. The EPILOGUE study is the first randomized controlled study to compare a combined approach with a stepwise approach for VT ablation in patients with ischemic cardiomyopathy.

### Limitations

This study is an open study that may subsequently lead to bias. However, the primary endpoint is recurrence of VT, an objective endpoint. Patients who have had a coronary artery bypass graft will be excluded from this study, thereby excluding a relevant part of the scar-related VT population. Open heart surgery is a known risk factor for adhesions and has shown to limit epicardial VT ablation [16]. When considering that VT ablation is not yet general practice, the inclusion rate may be low. This approach may change in following years as a consequence of improved understanding of the underlying pathophysiology or mechanism of VT.

## Trial status

Inclusion of eligible candidates in the EPILOGUE study has not yet started; enrolment is expected to start in May 2015.

## Abbreviation

VT: ventricular tachycardia
